# Systematic Review and Meta-Analysis of Randomized Controlled Trials (RCTs) Revealing the Future of Airway Management: Video Laryngoscopy vs. Macintosh Laryngoscopy for Enhanced Clinical Outcomes

**DOI:** 10.7759/cureus.50648

**Published:** 2023-12-17

**Authors:** Hany A Zaki, Eman Shaban, Mohamed Elgassim, Mohamed Fayed, Kaleem Basharat, Wael Elnabawy, Mohammed Gafar Abdelrahim, Ali Elkandow, Ahmed Mahdy, Aftab Azad

**Affiliations:** 1 Emergency Medicine, Hamad Medical Corporation, Doha, QAT; 2 Cardiology, Al Jufairi Diagnostic and Treatment, Doha, QAT; 3 Medicine, Taylor's University, Subang Jaya, MYS

**Keywords:** systematic review and meta-analysis, mac laryngoscopy, tracheal intubation, video laryngoscopy (vl), laryngoscopy

## Abstract

Since the 1940s, Macintosh laryngoscopy (Mac laryngoscopy) has been the gold standard for tracheal intubation, offering visualization of the glottis entrance. However, recent years have witnessed the emergence of various video laryngoscopy (VL) techniques. This systematic review and meta-analysis aims to assess the clinical outcomes of VL *versus* Mac laryngoscopy in an elective setting. We comprehensively searched five medical databases - PubMed, EMBASE, Medline, Cochrane Library, and Web of Science. All the databases were last searched in January 2023. We only included studies with full texts comparing VL to Mac laryngoscopy clinical outcomes. Studies were excluded if they were non-full text or non-randomized controlled trials (RCTs) and did not compare VL to Mac laryngoscopy. We extracted data comprising author names, publication year, key study outcomes (first-attempt intubation success rate, Cormack and Lehane grade, hypoxia incidence, and glottis view quality), video laryngoscope types, and sample sizes of both VL and Mac laryngoscopy groups. The Cochrane risk of bias tool was used to assess the risk of bias in the included studies. Statistical analysis was performed using Review Manager (RevMan, version 5.4; Cochrane Collaboration, London, UK), presenting results as odds ratio (OR) and risk ratios (RR) at a 95% confidence interval (CI). This facilitated the identification of relevant and appropriate studies of our analysis. The search produced 19 studies that were included in this review. The evaluated sample size ranges from 40 to 802, with 3,238 participants. The rate of success at the first attempt in the use of VL was 1,558/1,890 (82.43%), while the success rate for Mac laryngoscopy was 982/1,348 (72.85%; OR: 1.98 (1.25, 3.12)) at a 95% confidence interval. Pooled analysis indicated no significant difference for hypoxia concerning the type of device used RR (random effects: 1.02; 95% CI: 0.80-1.29). A video laryngoscope had a higher likelihood of visualizing the vocal cords categorized as category 1 in the Cormack-Lehane system of classification (RR: 2.45; 95% CI: 1.43-4.21). Additionally, considerably better glottis views were attained during VL than Mac laryngoscopy (OR: 1.77; 95% CI: 1.19-2.62). In elective tracheal intubation, VL demonstrates superior first-attempt success rates, offers improved glottis visualization, and reduces instances where the glottis cannot be viewed compared to Mac laryngoscopy.

## Introduction and background

Since the 1940s, when direct Macintosh laryngoscopy (Mac laryngoscopy) was introduced to ease tracheal intubation through visualization of the glottis entrance, it has advanced to be the standard equipment and technique for endotracheal intubation [1]. Notwithstanding the technological advancements in the medical field, tracheal intubation is performed using this old-fashioned method worldwide. It should be kept in mind that challenging, delayed, and unsuccessful intubation, in addition to "cannot intubate cannot ventilate" (CICV) scenarios, currently account for almost 39% of events during anesthesia [2]. Various types of video laryngoscopy (VL) have been invented in the last few years. The costs are considerably higher (for instance, Glide Scope costs between USD 6,500 and USD 7,000) than that of a Mac laryngoscopy (standard laryngoscope) [3].

Despite the considerable costs, all types of video laryngoscopes have related features. For instance, they comprise a grip and laryngoscope blade with a video camera attached at the blade's very end, combined with a light source. A liquid crystal display (LCD) video system exhibits the images. This allows video laryngoscopes to permit a broader visualizing angle; hence, the alignment of the pharyngeal, oral, and tracheal axes is unnecessary [4]. The outbreak of the coronavirus disease 2019 (COVID-19) pandemic has increased this interest [5] as COVID-19 airway recommendations suggest VL usage as a leading method in improving first pass success and allowing an increase of distance between the intubator and the patient's airway [6]. Moreover, VL does not need the alignment of the pharyngeal, oral, and tracheal axes. It also offers a better view of the glottis, which is easily mastered. The accomplishment of intubation relies on numerous factors such as design (Macintosh blade/acute angled; channeled/non-channeled), image quality on the monitor, illumination of the larynx/oropharynx, anatomy of the patient, previous history of intubation difficulty, emergency/elective intubation, experience and skills of the intubator, and the background setting (pre-hospital, in theatre or intensive care unit) [7].

VL has been widely used for tracheal intubation, especially during adverse conditions, due to the confirmed advantages of an improved success rate, an improved laryngeal view, and a fast learning curve [8]. Moreover, many specialists advocate for an extensive (and even exclusive) usage of VL [9]. Several randomized controlled trials (RCTs) comparing VL to conventional Mac laryngoscopy have been published. A good number of published clinical trials have permitted several reviews and meta-analyses. The reliability and quality of the results rely on the quality of the critical studies and if they are analogous enough to yield significant results when pooled [10]. For instance, combining hyperangulated VL (HA-VL) and Macintosh-VL (Mac-VL) trials into a pooled VL group might be unsuitable, considering the significant variances in techniques and indications between the blades. These meta-analyses could not identify the blade type to assist in the success of first-pass tracheal intubation. It is also worth noting that content capability is vital to decide if differences in practitioner experience, patients recruited, care context, clinical specialty, and location are too unlikely to be pooled in a meta-analysis. When significant heterogeneity is apparent, underlining such possible methodological flaws can advise forthcoming studies and aid in circumventing key confounders that avoid substantial inferences.

The current systematic review and meta-analysis aim to measure the clinical outcomes after using VL versus Mac laryngoscopy in an elective setting.

## Review

Methods

Protocol

This study was conducted according to the Preferred Reporting Items for Systemic Reviews and Meta-Analysis (PRISMA) [10]. PRISMA recommendations were observed in the inclusion and exclusion of studies, extraction of data, analysis, and result discussion to ensure the reliability of the results.

Search Strategy

An electronic search was performed on five databases: PubMed (RRID: SCR_004846), EMBASE (RRID: SCR_001650), Medline (RRID: SCR_002185), Cochrane Library (RRID: SCR_013000), and Web of Science (RRID: SCR_022706). The literature search was done in August 2022 by one investigator involved in the systematic review. A second party then confirmed their search results before submission for the inclusion and exclusion process. Four primary keywords, "direct laryngoscopy, video laryngoscopy, Macintosh laryngoscopy, and hyper angulated blade," were used to form the search queries. Additional usage of medical subject headings (MeSH), truncations, Boolean operators, and field tags was essential to increase the database search's precision. Besides searching in the electronic databases, other studies were identified manually through the lists of references.

Eligibility Criteria

Two independent authors participating in the current systematic review and meta-analysis reviewed the identified studies. The findings of this review were later merged through amicable discussions. A consensus was created through dispute resolutions when a contention of inclusion arose. The inclusion criteria focused on studies that measured the clinical outcomes after a patient underwent VL versus Mac laryngoscopy. Sufficiency of information was an essential factor beyond screening abstracts and titles. The previously engaged independent investigators conducted a full-text analysis to ensure that these studies' objectives and outcome measures matched this systematic review and meta-analysis. Priority was given to RCTs for inclusion, but cohort (CH) studies and method cohort (MCH) were also considered for inclusion due to their scarcity. However, RCTs compared either VL with a placebo or Mac laryngoscopy with a placebo. This systematic review and meta-analysis incorporated any study that fell into this category. To build a performance comparison, studies had to have reported the clinical outcome of the (type of laryngoscopy used. This clinical outcome measure created the basis of our interventional comparison. Time limits were not a limitation for eligibility but allowed the systematic review to include as many valuable studies as possible. Studies were excluded if they were non-full text or non-RCTs and did not compare VL to Mac laryngoscopy.

Risk of Bias Assessment

The risk of bias assessment tool (RoB 2) was utilized to evaluate the risks of bias in the included studies. Two reviewers working together assessed the domains to evaluate the risk of bias for all included studies. Five domains were considered using the instrument. The domains assessed had bias from randomization, deviations from the intended interventions, missing outcome data, measurement of the outcome, and bias in selecting the reported result.

Data Extraction

Two investigators extracted data from the included studies using a standardized form. Different views were resolved through group discussions. Data extracted included author, publication year, study outcomes (the successful rate at the first attempt of intubation, Cormack and Lehane, hypoxia and comparison of the glottis view), types of video laryngoscopes, and sample sizes of both VL and Mac laryngoscopy groups.

The Cochrane risk of bias tool was employed to evaluate the quality of studies and the degree of possible bias. The following domains were measured: allocation concealment sequence generation, personnel and outcome assessors' blinding of participants, selective outcome reporting, and incomplete data. It was impossible to blind the intubator to the intervention or the process measure assessors. However, there was feasibility in post-intervention outcome assessors to the type of device and blinding of patients.

Effect measures

Statistical Analysis

All statistical analysis was done using Cochrane Review Manager Version 5.4 (RevMan 5.4; Cochrane Collaboration, London, UK) (RRID: SCR_003581). All clinical outcomes measured were dichotomous outcomes (i.e., intubation success rate, hypoxia, improved visualization, and glottis view), so we calculated the odds ratios (OR) with a 95% confidence interval (CI). In pooling dichotomous data and computing pooled OR and risk ratio (RR) with 95% CIs, the Mantel-Haenszel technique was employed. We recorded several outcomes in small ordinal scales (i.e., intubation difficulty scores, improved visualization, and scales of the number of attempts) and converted these to dichotomous data wherever suitable. In multi-arm trials, a merged comparison group (merging all video laryngoscopes) compared with the standard group was used to form a sole pair-wise assessment. If it was impossible to combine data devoid of the error unit of analysis, we incorporated data from the video laryngoscope group, which could be next to offering a no-impact result.

We performed a meta-analysis of the outcomes with comparable measures of effect from two or more studies and where methodological, clinical, and statistical heterogeneity measures had a suitable pool of findings. The degree of statistical heterogeneity was classified using I^2^ statistics as per Choi et al. [11]. I^2^ values < 50% indicated less heterogeneity, and that above 75% was considered substantial. The selection of a random or fixed-effect statistical model for the meta-analysis was subjective to study characteristics, primarily the clinical or number of methodological differences between studies. For the success rate at the first attempt, a subgroup analysis based on the video device type was performed to explore clinical heterogeneity. Primarily, video laryngoscopes are categorized into those with a guiding channel and those that do not have a guiding channel [12]. For this review, they were classified into three groups: video laryngoscopes that have a guiding channel such as Airtraq; video laryngoscopes that have no guiding channel such as GlideScope; and other video laryngoscopes that do not have a guiding channel, such as McGrath Series 5, CEL-100, McGrath MAC, and C-MAC D-blade.

In the outcomes with more than 10 studies, funnel plots were visually inspected to estimate possible publication bias [13].

Results

Study Selection

**Figure 1:**
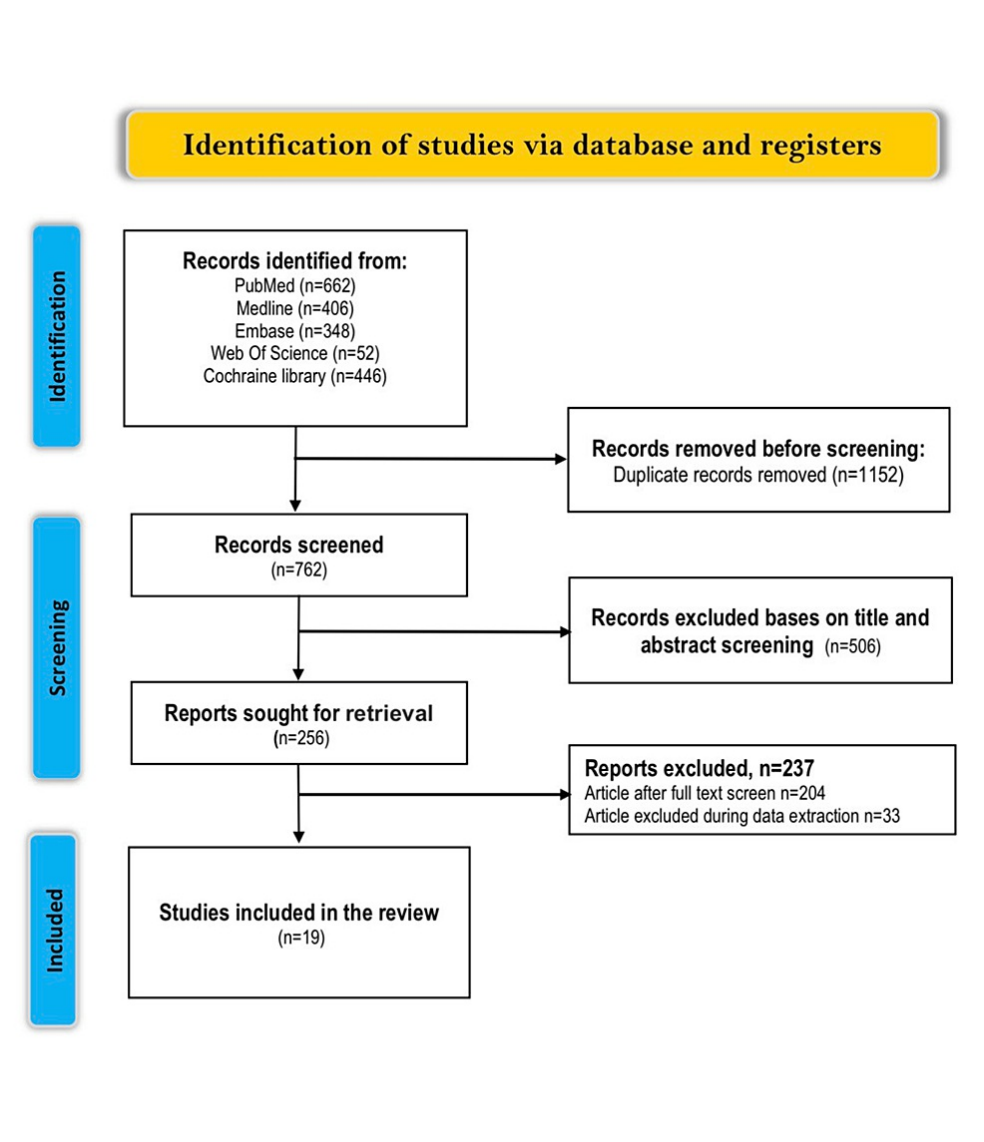
PRISMA flow diagram of the literature search results. PRISMA: Preferred Reporting Items for Systematic Reviews and Meta-Analyses

The search produced 1,914 studies that were screened and considered for inclusion. One thousand one hundred fifty-two studies were excluded due to duplication, and 762 were considered for a title and abstract screening. Based on the title and conceptual relevance, 506 studies were excluded. Of the 506 studies, 187 were systematic reviews and meta-analyses, 119 were commentaries, 72 were case reports, and 128 were supplement studies. The next stage was a full-text screening that looked at 256 studies and eliminated 204 since they were not full-text publications. A total of 15 studies were left for inclusion, with four additional studies identified in reference lists of former systematic reviews and meta-analyses, making a total of 19. Figure 1 shows the PRISMA diagram for the selection process.

Characteristics of the Included Studies

All studies were published between January 2008 and August 2022. The sample size ranges from 40 to 802, with 3,238 members. All the members were patients aged 16 years and above. All the studies included in this systematic review and meta-analysis were RCTs. The summary of the characteristics of the included studies is shown in Table 1. The significant features of the 19 included studies are also shown in the table.

**Table 1:** Summary of the characteristics of the included studies. RCT: Randomized controlled trial

Study	Study type	Sample size (VL/ML)	Intubation success rate (VL/ML)	Cormack-Lehane (VL/ML)	Glottis view (VL/ML)	Hypoxia (VL/ML)	Experience of the person performing laryngoscopy	Type of VL	Comparison
Ilyas et al., 2014 [14]	RCT	64/64	59/64				experienced	McGrath	direct
Roh et al., 2019 [15]	RCT	40/40	39/37	21/1	28/24		experienced	McGrath	direct
Sato et al., 2017 [16]	RCT	20/20	20/20	17/9	13/11		experienced	McGrath	direct
Aoi et al., 2010 [17]	RCT	18/18	17/17				experienced	Pentax AWS	direct
Kwak et al., 2016 [18]	RCT	35/35	35/35	29/25			experienced	McGrath	direct
Yoo et al., 2018 [19]	RCT	41/41	41/41	34/15			experienced	McGrath	direct
Russell et al., 2013 [20]	RCT	35/35	29/32	10/28			experienced	GlideScope	direct
Wasem et al., 2013 [21]	RCT	30/30	28/26				experienced	Airtraq	direct
Lakticova et al., 2013 [22]	RCT	252/140	199/75			30/9	experienced	GlideScope	direct
Taylor et al., 2012 [23]	RCT	44/44	44/26	42/11	41/28		experienced	McGrath	direct
Kim et al., 2013 [24]	RCT	22/23	22/23	22/0			experienced	Pentax AWS	direct
Shah et al., 2016 [25]	RCT	30/29	26/16				experienced	C MAC	direct
Ranieri et al., 2014 [26]	RCT	68/64	68/54				experienced	Airtraq	direct
Ndoko et al., 2008 [27]	RCT	53/53	53/47				experienced	Airtraq	direct
Aziz et al., 2012 [28]	RCT	149/147	138/124			8/7	experienced	C MAC	direct
Hypes et al., 2016 [29]	RCT	673/136	541/89			123/35	experienced	GlideScope	direct
Lascarrou et al., 2017 [30]	RCT	186/185	126/130		112/92	20/20	experienced	McGrath	direct
Janz et al., 2016 [31]	RCT	70/140	55/96		32/48	14/16	experienced	GlideScope	direct
Frohlich et al., 2011 [32]	RCT	60/100	18/30	29/21			experienced	McGrath	direct

Risk of Bias of the Included Studies

**Figure 2:**
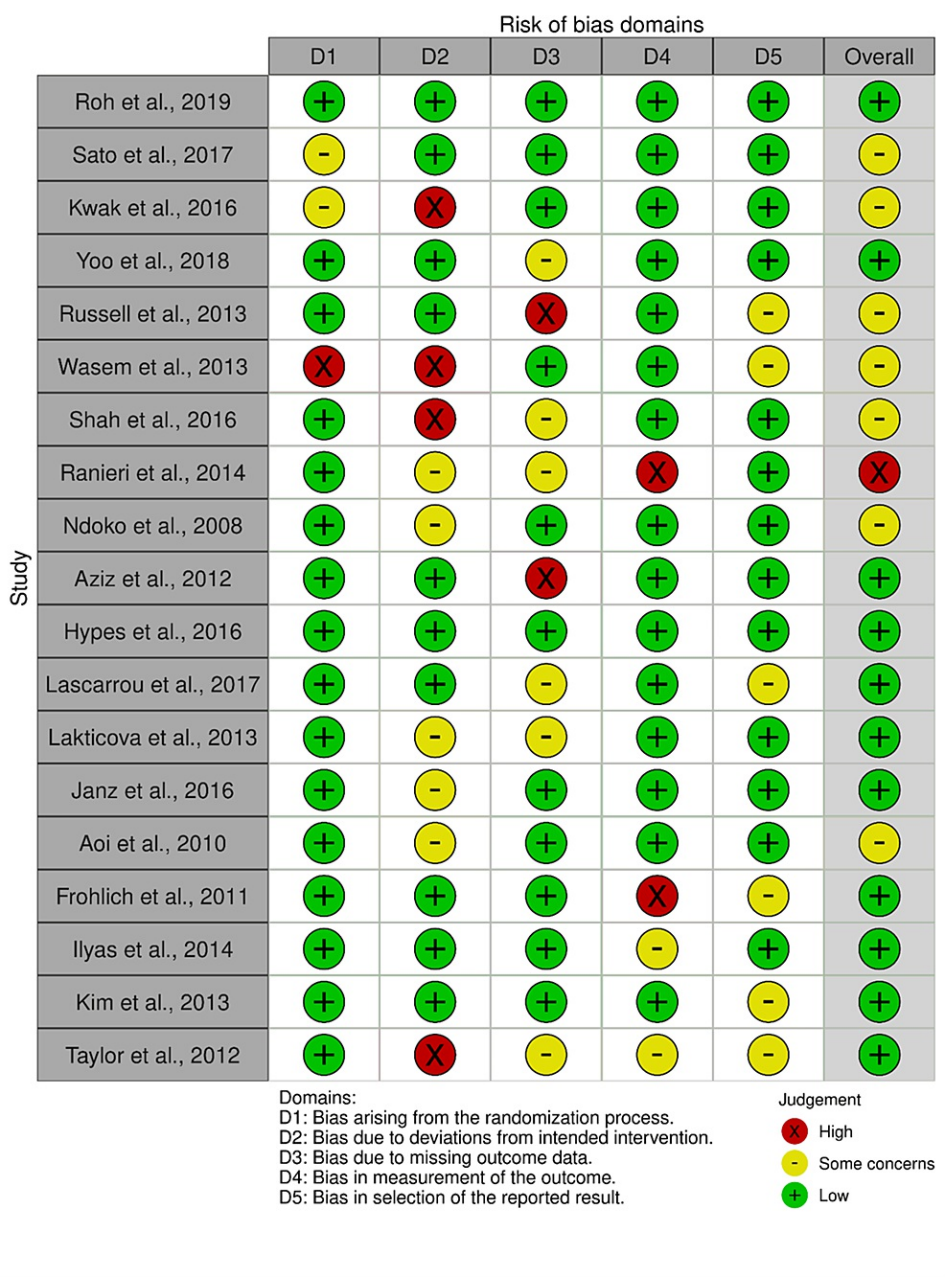
Risk of bias graph. Authors' judgments on each risk of bias item. Ilyas et al., 2014 [14], Roh et al., 2019 [15], Sato et al., 2017 [16], Aoi et al., 2010 [17], Kwak et al., 2016 [18], Yoo et al., 2018 [19], Russell et al., 2013 [20], Wasem et al., 2013 [21], Lakticova et al., 2013 [22], Taylor et al., 2012 [23], Kim et al., 2013 [24], Shah et al., 2016 [25], Ranieri et al., 2014 [26], Ndoko et al., 2008 [27], Aziz et al., 2012 [28], Hypes et al., 2016 [29], Lascarrou et al., 2017 [30], Janz et al., 2016 [31], Frohlich et al., 2011 [32]

All the studies were randomized, with 12 studies giving enough details of the met randomization methods. The risk of bias graph is presented in Figure 2.

**Figure 3:**
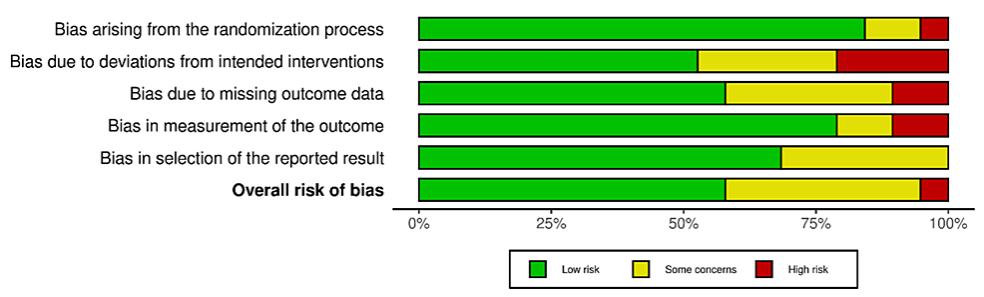
Risk of bias summary plot.

The risk of bias summary plot of the included studies is presented in Figure 3.

Statistical results

Primary Outcome: Intubation Success Rate

**Figure 4:**
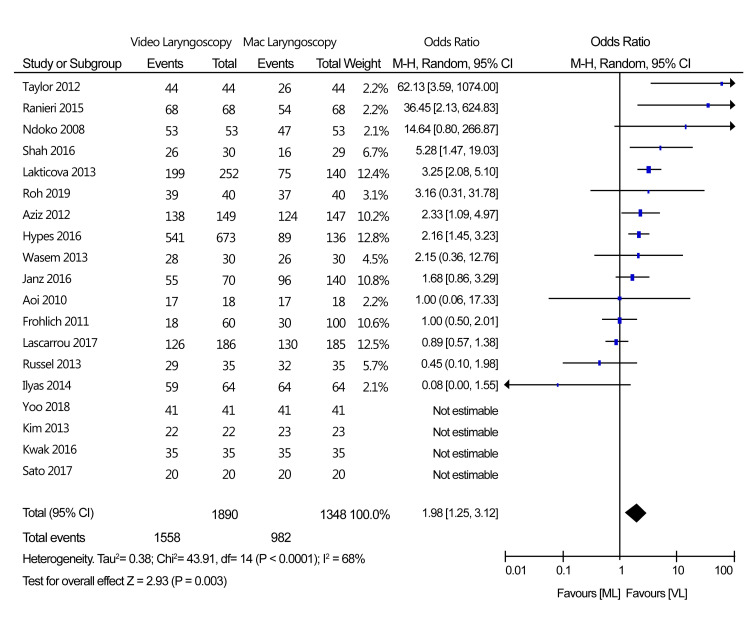
Forest plot of the rate of success at the first attempt (VL vs. Mac laryngoscopy). Ilyas et al., 2014 [14], Roh et al., 2019 [15], Sato et al., 2017 [16], Aoi et al., 2010 [17], Kwak et al., 2016 [18], Yoo et al., 2018 [19], Russell et al., 2013 [20], Wasem et al., 2013 [21], Lakticova et al., 2013 [22], Taylor et al., 2012 [23], Kim et al., 2013 [24], Shah et al., 2016 [25], Ranieri et al., 2014 [26], Ndoko et al., 2008 [27], Aziz et al., 2012 [28], Hypes et al., 2016 [29], Lascarrou et al., 2017 [30], Janz et al., 2016 [31], Frohlich et al., 2011 [32] VL: Video laryngoscopy; Mac laryngoscopy: Macintosh laryngoscopy

All 19 included studies reported an intubation success rate as an outcome. According to the meta-analysis of the 19 trials, the rate of success of tracheal intubation using a VL was considerably higher than that of Mac laryngoscopy: 1,558/1,890 (82.43%) success rate for VL and 982/1,348 (72.85%) success rate for Mac laryngoscopy. The effect size of this clinical outcome of the two types of devices was 1.98 (OR: 1.25, 3.12) at a 95% CI. A test for the overall effect of the two treatment options was Z = 2.93 (p < 0.0001). However, the included studies were moderately heterogeneous, with the I^2^ = 68%. The moderate heterogeneity can be attributed to the various periods during which individual studies measured the intubation success rate. Statistical results of this analysis are represented in a forest plot, and publication bias is described in Figure 4.

**Figure 5:**
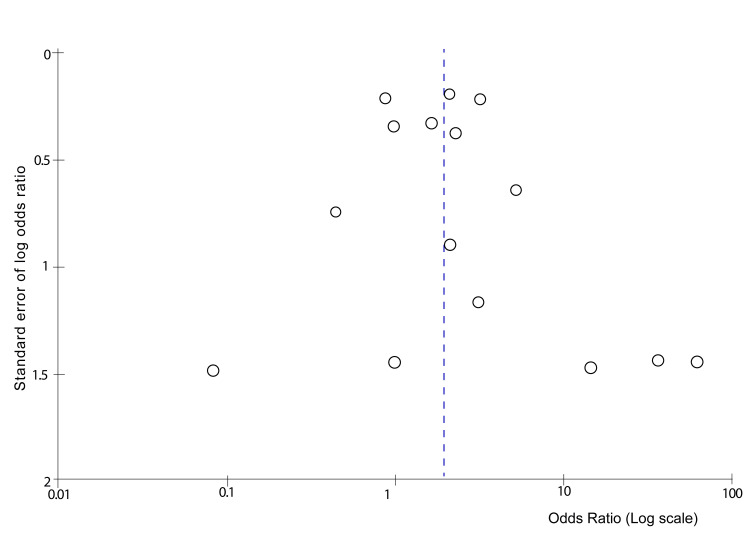
A funnel plot analysis for publication bias on the primary outcome.

A funnel plot analysis for publication bias on the primary outcome was described in Figure 5.

The funnel plot indicates the presence of publication bias due to its asymmetrical nature.

Secondary outcomes

Hypoxia

**Figure 6:**
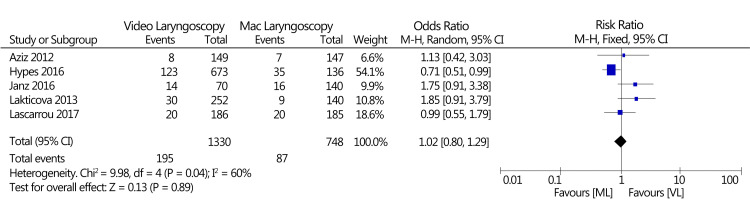
The forest plot of the occurrences with hypoxia (VL vs. Mac laryngoscopy). VL: Video laryngoscopy; Mac laryngoscopy: Macintosh laryngoscopy Lakticova et al., 2013 [22], Aziz et al., 2012 [28], Hypes et al., 2016 [29], Lascarrou et al., 2017 [30], Janz et al., 2016 [31]

Eight studies informed hypoxia, and only five had event data. A pooled analysis indicated no significant differences in hypoxia based on the type of device used RR, random-effects 1.02, 95% CI (0.80-1.29), I^2^ = 60%; test for overall effect Z = 0.13; and p = 0.89. Statistical analysis of this outcome is indicated in Figure 6.

Cormack-Lehane

**Figure 7:**
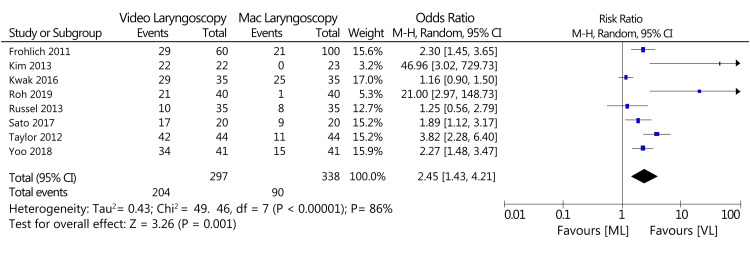
A forest plot of the Cormack–Lehane system of classification (VL vs. Mac laryngoscopy). VL: Video laryngoscopy; Mac laryngoscopy: Macintosh laryngoscopy Roh et al., 2019 [15], Sato et al., 2017 [16], Kwak et al., 2016 [18], Yoo et al., 2018 [19], Russell et al., 2013 [20], Taylor et al., 2012 [23], Kim et al., 2013 [24], Frohlich et al., 2011 [32]

During intubation, when using a video laryngoscope, there was a higher degree of getting a view of vocal cords, which was categorized as category 1 in the Cormack-Lehane system of classification (RR: 2.45; 95% CI: 1.43-4.21; n = 635; test for overall effect Z = 3.26; p = 0.001; I^2^ = 86%. A forest plot for the statistical analysis of this outcome is represented in Figure 7.

Glottis View

**Figure 8:**
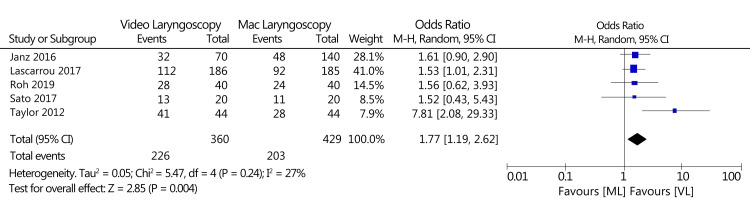
A forest plot of the glottis view as an outcome (VL vs. Mac laryngoscopy). VL: Video laryngoscopy; Mac laryngoscopy: Macintosh laryngoscopy Roh et al., 2019 [15], Sato et al., 2017 [16], Taylor et al., 2012 [23], Lascarrou et al., 2017 [30], Janz et al., 2016 [31]

Five of the 19 trials compared the glottis view between VL and Mac laryngoscopy in intubation. Considerably improved glottis views were attained in VL compared to Mac laryngoscopy in all of the five trials (OR: 1.77; 95% CI: 1.19-2.62; n = 789; overall effect test Z = 2.85; p = 0.004; I^2^ = 27%). The statistical results for this outcome are demonstrated by a forest plot in Figure 8.

The statistical analysis of success rate, hypoxia, Cormack-Lehane, and glottis review is summarized in Table 2.

**Table 2:** Summary of the statistical analysis of the success rate, hypoxia, Cormack-Lehane, and glottis view.

Clinical outcome	Number of trials	RR or OR 95% CI	p-Value	Cochrane's Q	I^2^ statistic, %
Success rate	19	1.98 [1.25, 3.12]	<0.0001	43.91	68
Hypoxia	5	1.02 [0.80, 1.29]	0.04	9.98	60
Cormack-Lehane	8	2.45 [1.43, 4.21]	<0.00001	49.46	86
Glottis view	5	1.77 [1.19, 2.62]	0.24	5.47	27

Subgroup analysis

**Figure 9:**
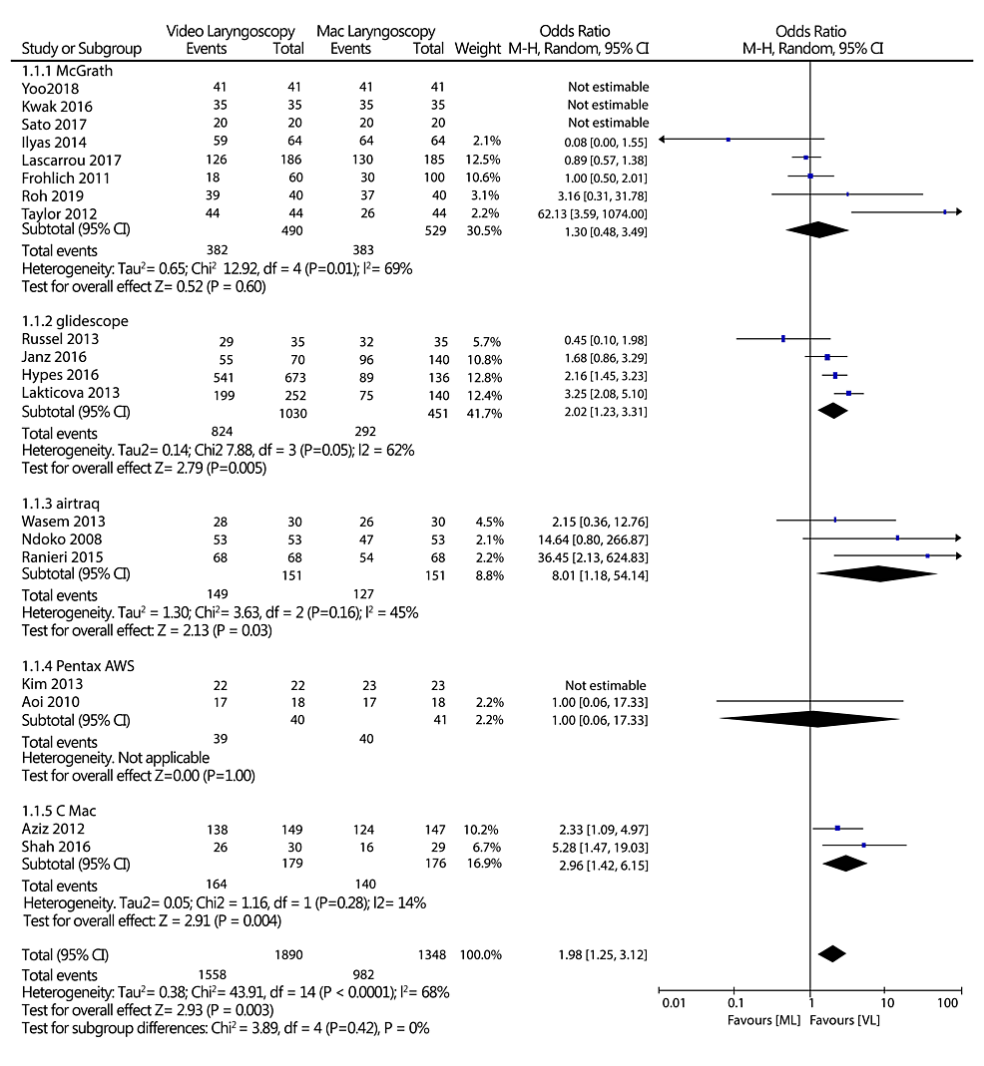
A forest plot for the subgroup analyses based on the video laryngoscopy type (VL vs. Mac laryngoscopy). VL: Video laryngoscopy; Mac laryngoscopy: Macintosh laryngoscopy Ilyas et al., 2014 [14], Roh et al., 2019 [15], Sato et al., 2017 [16], Aoi et al., 2010 [17], Kwak et al., 2016 [18], Yoo et al., 2018 [19], Russell et al., 2013 [20], Wasem et al., 2013 [21], Lakticova et al., 2013 [22], Taylor et al., 2012 [23], Kim et al., 2013 [24], Shah et al., 2016 [25], Ranieri et al., 2014 [26], Ndoko et al., 2008 [27], Aziz et al., 2012 [28], Hypes et al., 2016 [29], Lascarrou et al., 2017 [30], Janz et al., 2016 [31], Frohlich et al., 2011 [32]

For the primary outcome, the success rate at the first attempt, a subgroup analysis was performed based on the types of VL and the comparison with Mac laryngoscopy. In this subgroup analysis, McGrath, GlideScope, Airtraq, and C Mac types of VL showed superiority compared to Mac laryngoscopy's success rate during the first attempt. By contrast, the Pentax AWS VL type indicated no significant differences compared to Mac laryngoscopy's success rate at the first attempt (GlideScope: OR, random-effects 2.02, 95% CI: 1.23-3.321, I^2^ = 62%, n = 1481; Pentax AWS: OR, random effects 1.00, 95% CI 0.06 to 17.33; n=81, McGrath OR, random-effects: 1.30, 95% CI 0.48 to 3.49; I2 =69%; n=1019, C Mac: OR, random effects: 2.96, 95% CI: 1.42-6.15, I^2^ = 14%, n = 355; Airtraq: OR, random effects: 8.01, 95% CI: 1.18-54.14, I^2^ = 45%, n = 302) (Table 3). As shown in the forest plot in Figure 9, the test for subgroup differences suggested that no statistically significant subgroup effect was present (p = 0.42), meaning that the type of VL does not statistically significantly modify the effect of VL compared to Mac laryngoscopy. Furthermore, preliminary trials (less than five) and participants (less than 1,800) were included in all subgroups, except McGrath subgroups, so the covariate distribution is concerning in this subgroup analysis. A summary of the subgroup analysis for the comparison results is presented in Table 3.

**Table 3:** Summary of the subgroup analysis of the VL vs. ML comparison.

Subgroup	Number of studies	VL vs. ML OR (95% CI)	Heterogeneity (I^2^) (%)	P-value for the heterogeneity
McGrath	8	1.30 [0.48, 3.49]	69	0.01
GlideScope	4	2.02 [1.23, 3.321]	62	0.05
Airtraq	3	8.01 [1.18, 54.14]	45	0.16
C Mac	2	2.96 [1.42, 6.15]	14	0.28
Pentax AWS	2	1.00 [0.06, 17.33]	Not applicable	Not applicable

A forest plot for the subgroup analysis of this comparative study is shown in Figure 9.

Discussion

Nineteen epidemiological studies comparing VL and Mac laryngoscopy met the inclusion criteria. The most important findings of this meta-analysis are as follows: (i) the rate of success at the first attempt after the use of VL was significantly higher than that of Mac laryngoscopy: 1,558/1,890 (82.43%) rate of success at the first attempt for VL and 982/1,348 (72.85%) success rate at the first attempt for Mac laryngoscopy; (ii) there is no significant difference in hypoxia after using VL versus Mac laryngoscopy; (iii) there was an improved probability of getting a view of the vocal cords that were categorized as category 1 in the Cormack-Lehane system of classification after using VL compared with Mac laryngoscopy; and (iv) considerably improved glottis views were attained in VL compared with Mac laryngoscopy. Specifically, this research reinforces the growing preference for VL over traditional methods in airway management, aligning with the trend in current medical literature toward adopting advanced technological approaches for enhanced patient safety and procedural efficacy.

The difference in rate of success of almost 10% at the first attempt might not look like much. However, the complication risks increase if additional intubation attempts are made [33]. The risk of advancement to a cannot intubate cannot ventilate (CICV) situation increases if an attempt at tracheal intubation is repeated [5]. The American Society of Anesthesiologists Closed Claims Project (ASA, CCP) analyses suggest an increase in mortality and hypoxic brain injury in such situations [34]. When Mac laryngoscopy is not successful, additional attempts using the same method can lead to an 80% rate of failure. By contrast, alternate methods such as VL will likely be successful [33]. The Society of Difficult Airway's guiding principles on the significance of first-time success are crystal clear. It is essential to capitalize on the success rate of the first attempt [1]. Saving the usage of a video laryngoscope for the next attempt wastes the first attempt and consequently makes the next one more challenging. The device's performance varies between handlers and devices and should be noted [35]. Marshall et al. [36] argued that clinics would most likely want to offer a variety of video laryngoscopes to provide their anesthetists with an option to pick the most appropriate device. The available variety will be limited due to prices; nevertheless, departments of anesthesiology might be harshly judged when a dangerous airway event occurs and is investigated in light of new guidelines [36]. Our results show consensus with previous systematic reviews and meta-analyses [37,38] showing that this improvement is more noticeable in patients that have a complex airway [39] and that endorse the usage of video laryngoscopes to accomplish successful intubation in patients that have a greater complex laryngoscopy risk [40].

Five studies reported results we managed to pool for hypoxia [22,28-31]. For this outcome, no significant difference in hypoxia after using VL compared to Mac laryngoscopy was observed. In this particular outcome, the findings are supported by a previous Cochrane systematic review, a comparative study of VL versus Mac laryngoscopy reviewing data from 1,319 participants, and a substantially smaller evidence base than that in the current meta-analysis [41].

The results of this review also indicated an increase in the degree of classification from the Cormack-Lehane classification system after using VL as compared to Mac laryngoscopy, which recommends the visualization of the glottis using the VL technique. This finding is also supported by another systematic review and meta-analysis [37].

The percentage of glottis opening (POGO) scores after optimal external laryngeal maneuvers reached 100% in the VL group, which was significantly higher than that in the Mac laryngoscopy group (94.8%). However, the two groups had no significant difference concerning desaturation and traumatic injuries [42].

Video laryngoscopes are anticipated to give an enhanced glottis view without the requirement to align tracheal, pharyngeal, and oral axes. In the current analysis, VL leads to better visualization of the glottis [15,16,23,30,31]. The number of laryngoscopies leading to a Cormack-Lehane grade 3 or 4 was fewer when VL was used in general and video laryngoscopes that have acutely angled blades precisely such as Pentax AWS, Airtraq, and GlideScope, as well as C-Mac. Video laryngoscopes with an acutely angled blade need an additional method for intubation. The blade shape adheres to the natural oral cavity anatomy, and the camera at the blade's tip brings the operator's point of view very near to the glottis. Various factors show that a flawless view does not always lead to smooth intubation [43]. Only two trials in the current systematic review evaluated C-Mac-shaped video laryngoscopes [25,31]. Due to only two studies, it was challenging to conclude anything comparing VL with Mac-shaped blades to video laryngoscopes with acutely angled blades. Seemingly, the finest view of the glottis is not always linked with the maximum success rate of first intubation. The findings in this outcome are consistent with previously performed meta-analyses [44].

Strengths and limitations

One of the key strengths of this systematic review and meta-analysis is that VL provides added value for an experienced anesthetist. In our experience, anesthetists experienced with Mac laryngoscopy occasionally appear reluctant to use video laryngoscopes. It is difficult to change old habits [45]. Undeniably, familiarity with Mac laryngoscopy does not translate to proficiency with VL, and being an expert in using one video laryngoscope does not automatically brand one an expert of all video laryngoscopes. Cooper et al. stated that explaining VL to skilled Macintosh users was more demanding than to beginners [46]. In their study, Cortellazzi et al. [47] showed that VL is a multifaceted skill that needs rigorous practice to attain expertise, including those skilled in Mac laryngoscopy. This means that, since VL has added value for skilled anesthetists, they should also train rigorously and must not depend on their proficiency with Mac laryngoscopy. Recently, Lewis et al., a Cochrane review, compared Mac laryngoscopy to VL for tracheal intubation [41]. Even though there are resemblances between the current study and their review, there are some significant differences. Namely, (1) the studies we reviewed had patients with an alleged airway difficulty, while the Cochrane review encompassed studies that use VL for tracheal intubation generally; (2) in this study, we only reviewed RCTs analyzing the intubation in adults that use VL and Mac laryngoscopy; and (3) studies concerning a simulated difficult airway were excluded, in contrary to the Cochrane review.

Lastly, this systematic review has several limitations. First, the studies included had a different strategy, study protocol, and endpoint, implying that measurement biases were present in our primary and secondary outcomes analyses. Secondly, most registered participants were adults; thus, these findings cannot be applied directly to pediatric populations. Furthermore, three trials presented conflicting recommendations that the Macintosh laryngoscope gave a higher intubation success rate in the first attempt [14,20,30]. Thirdly, tracheal intubation was frequently done in patients with expected difficult airways; nevertheless, this analysis categorized the cases as ordinary airways. Given this, the inferences would be problematic to apply to the expected airways. Lastly, two issues reduce the evidence quality of our outcomes: the first one is the impracticality of blinding due to the obvious visual differences of the two-intubating equipment, and the second one is the heterogeneity of the included studies.

## Conclusions

In conclusion, this comprehensive systematic review and meta-analysis of 19 epidemiological studies comparing VL and Mac laryngoscopy for adult tracheal intubation have yielded several significant findings. Firstly, VL demonstrated a significantly higher rate of first-attempt success than Mac, which is crucial given the increased risks associated with multiple intubation attempts. Secondly, there was no significant difference in the occurrence of hypoxia between VL and Mac, indicating the safety of both methods in this regard. Thirdly, VL consistently provided an improved view of the vocal cords, as evidenced by an increased probability of achieving a Cormack-Lehane classification of 1, further supporting its efficacy. Additionally, VL demonstrated a superior glottis view compared to Mac, with a higher percentage of POGO score, emphasizing its potential advantage in difficult airway situations. While there are strengths in terms of the added value of VL for experienced anesthetists, this study acknowledges the need for rigorous training. It emphasizes that proficiency in Mac laryngoscopy does not automatically transfer to VL. However, limitations include variability in study protocols, potential measurement biases, limited applicability to pediatric populations, conflicting recommendations in a few trials, and the inherent challenges of blinding due to visual differences between the two methods. Nonetheless, these findings collectively support the preference for VL over Mac in various clinical scenarios, especially in cases of complex airways, while recognizing the need for further research to address the identified limitations and provide more conclusive evidence.
